# Neuro-fuzzy controller to navigate an unmanned vehicle

**DOI:** 10.1186/2193-1801-2-188

**Published:** 2013-04-27

**Authors:** Boumediene Selma, Samira Chouraqui

**Affiliations:** Department of Computer Science, Faculty of Science, University of Science and Technology “Mohamed Boudiaf” USTO Oran, Oran, BP1505, Algeria

**Keywords:** Unmanned vehicle, Neural network, Fuzzy logic, ANFIS, Control

## Abstract

A Neuro-fuzzy control method for an Unmanned Vehicle (UV) simulation is described. The objective is guiding an autonomous vehicle to a desired destination along a desired path in an environment characterized by a terrain and a set of distinct objects, such as obstacles like donkey traffic lights and cars circulating in the trajectory. The autonomous navigate ability and road following precision are mainly influenced by its control strategy and real-time control performance. Fuzzy Logic Controller can very well describe the desired system behavior with simple “if-then” relations owing the designer to derive “if-then” rules manually by trial and error. On the other hand, Neural Networks perform function approximation of a system but cannot interpret the solution obtained neither check if its solution is plausible. The two approaches are complementary. Combining them, Neural Networks will allow learning capability while Fuzzy-Logic will bring knowledge representation (Neuro-Fuzzy). In this paper, an artificial neural network fuzzy inference system (ANFIS) controller is described and implemented to navigate the autonomous vehicle. Results show several improvements in the control system adjusted by neuro-fuzzy techniques in comparison to the previous methods like Artificial Neural Network (ANN).

## Introduction

Traditional control of non-linear dynamical systems has been done by using Classical Linear Control Theory and assuming simple linear mathematical models for the systems. However, it is now well known that non-linear dynamical systems can exhibit complex behavior (and as a consequence are difficult to control) and the most appropriate mathematical models for them are the non-linear ones. Since the complexity of mathematical models for real dynamical systems is very high it becomes necessary to use more advanced control techniques. This is precisely the fact that motivated researchers in the area of Artificial Intelligence (AI) to apply techniques that mimic human experts in the domain of dynamical systems control. More recently, techniques like neural networks and fuzzy logic have been applied with some success to the control of non-linear dynamical systems for several domains of application.

In literature, there can be found many different approaches related to the autonomous control of Unmanned vehicles UVs; some of the techniques proposed include fuzzy control (Banks & Hayward, 2001; Doitsidis, Valavanis, & Tsourveloudis, Doitsidis, Valavanis, & Tsourveloudis, ; Doitsidis, Valavanis, & Tsourveloudis, 2004; Verbruggen, Zimmerman, & Babuska, Verbruggen, Zimmerman, & Babuska, ; Verbruggen, Zimmerman, & Babuska, 1999), adaptive control (Andrievsky & Fradkov, 2002; Aström & Wittenmark, 1989; Schumacher & Kumar, 2000), neural networks (Sundararajan, Li, & Sratchandran, 2001), genetic algorithms (Cordon, Gamide, Herrera, & Magdelen, 2004) and Lyapunov theory (Ren & Beard, 2003).

However, there have been some limitations and problems with these approaches when applied to real systems. For this reason, we proposed in this paper the application of a hybrid approach for the problem of control, combining neural and fuzzy technologies called Adaptive neuro fuzzy inference system (ANFIS).

The ANFIS model was trained with the back propagation gradient descent method. We used the data described in (Andrzejak et al. 2001), which is publicly available. After learning of ANFIS in several iterations there is an error between the calculated position and the position of the reference which is minimal.

The correct training rates and convergence rates of the proposed ANFIS model were examined and then performance of the ANFIS model was reported in the results obtained.

The paper is organized as follows. Section 2 starts with a basic introduction of ANFIS and then explains the design of the controllers used for the autonomous control of the UAV. The inputs and the outputs of each controller are described and the membership functions used are given. The hybrid learning algorithm adopted in this work is described in Section 3 and a representative simulation study and its results are presented in Section 4. In the final sections of the paper some concluding remarks and suggestions for future work are made.

## Overview of the ANFIS

Adaptive Neural Fuzzy Inference System (ANFIS) is fuzzy Sugeno model put in the framework to facilitate learning and adaptation procedure. Such network makes fuzzy logic more systematic and less relying on expert knowledge. The objective of ANFIS is to adjust the parameters of a fuzzy system by applying a learning procedure using input–output training data.

A combination technique of least square algorithm and back propagation are used for training fuzzy inference system (Jang & Sun, 1997).

**Figure 1:**
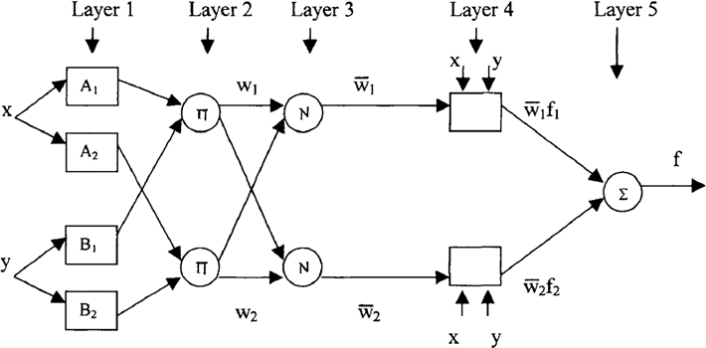
**Equivalent ANFIS architecture (adaptive nodes shown with square and fixed nodes with a circle).**

Basic architecture of ANFIS that has two inputs x and y and one output f is shown in Figure 1. Assume the rule base contains two Takagi-Sugeno if-then rules as follows:

- Rule 1: If *x* is *A*_*l*_ and *y* is *B*_*1*_, then *f*_*l*_*= p*_*l*_*x + q*_*l*_*y + r*_*l,*_
- Rule 2: If *x* is *A*_*2*_ and *y* is *B*_*2*_, then *f*_*2*_*= p*_*2*_*x + q*_*2*_*y + r*_*2*_*,*

ANFIS has five layers as shown in Figure 1, square nodes are named adaptive nodes, to demonstrate that the parameters in these nodes are adjustable., while circle nodes, named fixed nodes, are to demonstrate that the Parameters are fixed. Then the node function in each layer is described below:

Layer 1: in the first layer, all the nodes are adaptive nodes. The outputs of layer 1 are the fuzzy membership grade of the inputs, every node *i* in this layer is an adjustable node with node function:12

Where ,  can adopt any fuzzy membership function. For example, if the bell shaped membership function is employed,  is given by:3

Where *a*_*i*_, *b*_*i*_ and *c*_*i*_ are the parameters of the membership function, governing the bell shaped functions accordingly.

Layer 2: in the second layer, the nodes are fixed nodes. They indicate that they perform as a simple multiplier.

The outputs of this layer can be represented as:4

Which are the so-called firing strengths of the rules.

Layer 3: in the third layer, the nodes are also fixed nodes. They indicating that they play a normalization role to the firing strengths from the previous layer.

The outputs of this layer can be represented as:5

Which are the so-called normalized firing strengths?

Layer 4: in the fourth layer, the nodes are adaptive nodes. The output of each node in this layer is simply the product of the normalized firing strength and a first order polynomial (for a first order Sugeno model). Thus, the outputs of this layer are given by:6

Layer 5: in the fifth layer, there is only one single fixed node. This node performs the summation of all incoming signals. Hence, the overall output of the model is given by:7

It can be observed that there are two adaptive layers in this ANFIS architecture, namely the first layer and the fourth layer. In the first layer, there are three modifiable parameters {*a*_*i*_, *b*_*i*_, *c*_*i*_}, which are related to the input membership functions. These parameters are the so-called premise parameters. In the fourth layer, there are also three modifiable parameters {*pi*, *qi*, *r*_*i*_}, pertaining to the first order polynomial. These parameters are so-called consequent parameters (Jang, 1993).

## System design

The efficacy of the ANFIS controllers is evaluated by demanding the UV to execute some steering maneuvers autonomously.

**Figure 2:**
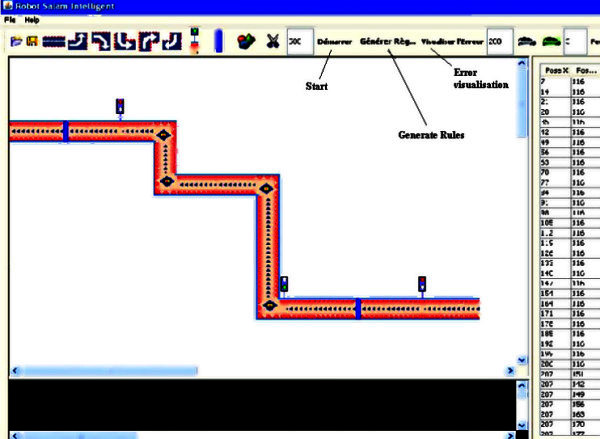
**Reference path.**

**Figure 3:**
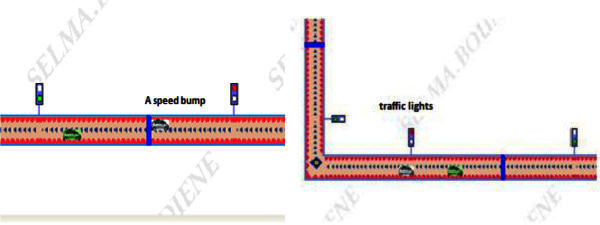
**The obstacles Maneuver with UV.**

There are some basic maneuvers described in the road literature. One of them is steep turns. Steep turns aim to see the domination of the pilot over the control surfaces of the plane in basic training. There are several types of steep turn maneuvers. The one more that is used here is the case of precession of others cars on the road that have minimum speeds to that of UV which starts precession operation by making horns from 3 meters taking the second position of the road, as shown in Figures 2 and 3.

Road reference also contains bumps and traffic lights, it should here be noted that while the UV is in front of a bumps must slow up, and if there is a traffic lights, the nose of the UV is placed at line of traffic lights if it is red, if not it will finish without stopping and with the same speed.

The rules of the neuro-fuzzy inference subsystem are formulated so that all possible potential situations that may occur to the vehicle on the route are taken into consideration.

Table 1 presents the different situations and fuzzy rules, they are translated.

**Table 1:** **ANFIS situations**

Situation	Antecedent	Consequent
***Case 1: donkey***		
Distance donkey “D”	D = 10 m	Decrease the speed
	D > 10 m	**/**
***Case2: existence of a vehicle on the road***		
Distance from another vehicle on the road “D-V”	D_V =3 m	Change of position and Exceeding
	D_V >3 m	**/**
***Case 3: traffic lights***		
Color traffic lights “C”	C = Green	Move
	C = Red	Stop

## The neuro-fuzzy architecture

The input layer passes the data to the second layer, which calculates the fuzzy membership degrees to which the input values belong to predefined fuzzy membership functions. The third layer contains fuzzy rule nodes representing prototypes of input–output data as an association of hyper-spheres from the fuzzy input and fuzzy output spaces. Each rule node is defined by 2 vectors of connection weights, which are adjusted through the hybrid learning technique. The fourth layer calculates the degrees to which output membership functions are matched by the input data, and the fifth layer does defuzzification and calculates exact values for the output variables.

**Figure 4:**
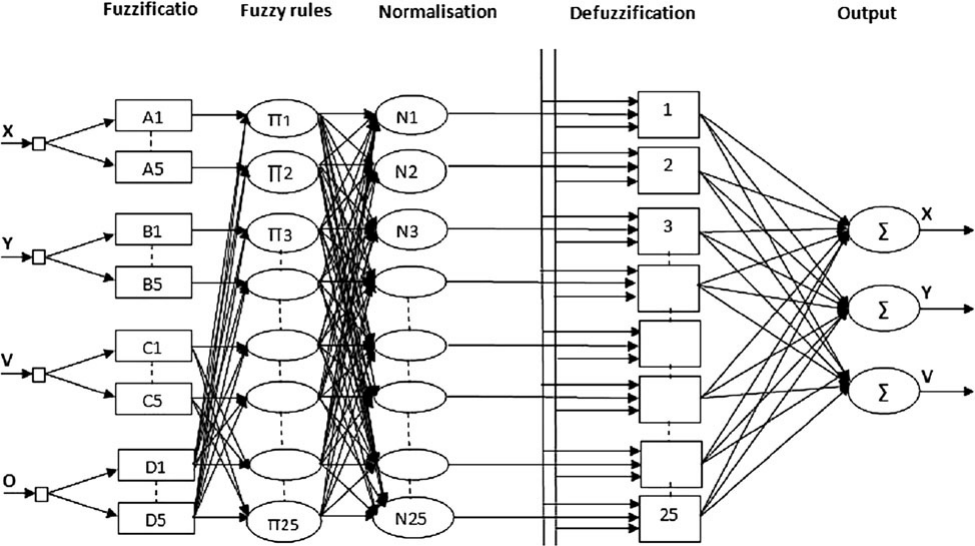
**Neuro-fuzzy Architecture.**

As it is illustrated in Figure 4, at the beginning of the simulation, we inject the fuzzy knowledge base (Takagi Sugeno coefficient) on the network controller, we give result in an entry (the initial positions of the reference route *(X, Y)*, the velocity *V*, back to donkey *D, F* red light, vehicle *O* which shows the state of overshoot) and three outputs (new positions *(X, Y)*, and the velocity *V*).

According to the calculations of the network (fuzzification, inference, defuzzification) the control action is applied to the input of the process that lets you know the state of the system to calculate the error which has the input of the network controller.

The different rules used on the system are summarized as follow:

**Figure 5:**
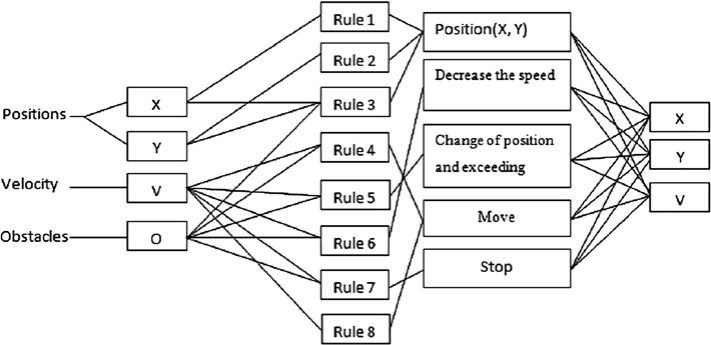
**ANFIS rules configuration.**

- Rule 1: IF Position *X* is *X*^*’*^ and *X-ϵ <X < X+ϵ* THEN the Position is *X*
- Rule 2: IF Position *Y* is *Y*^*’*^ and *Y-ϵ <Y < Y+ϵ* and THEN the Position is *Y*
  Where *ϵ* defined the membership interval of the output calculated relative to the desired output and it is empirically determined by the user.
- Rule 3: IF Position is *X, Y* and Obstacle is Null THEN the Position is *X, Y*
- Rule 4: IF Velocity MAX and Obstacle is Null THEN the Velocity is MAX
- Rule 5: IF Obstacle is Vehicle and Velocity is MAX THEN the Position is LEFT and exceed
- Rule 6: IF Obstacle is donkey and Velocity is max THEN the Velocity is MIN
- Rule 7: IF Obstacle is traffic lights and Color is RED THEN the Velocity is ZERO
- Rule 8: IF Offset is traffic lights and Color is GREEN THEN the Velocity is MAX
- The value at the end of each rule represents the initial weight of the rule, and will be adjusted to its appropriate level at the end of training. All the rules lead to three different subjects, which is the steer direction for the mobile robot and velocity. Then three output nodes are needed. They are Positions *X, Y* and Velocity correspondingly.

The Figure 5 illustrates the ANFIS rules configuration.

## Training for neuro-fuzzy system

The weight for each neural node is configured with an initial value specified by system experts, and then further tuned by using a training algorithm. A back propagation algorithm is employed in this research as follows:

- Step 1: Present an input data sample, compute the corresponding output
- Step 2: Compute the error between the output(s) and the actual target(s)
- Step 3: The connection weights and membership functions are adjusted
- Step 4: At a fixed number of epochs, delete useless rule and membership function nodes, and add in new ones
- Step 5: IF Error > Tolerance THEN goto Step 1 ELSE stop.

When the error level drops to below the user-specified tolerance, the final interconnection weight reflects the changes in the initial fuzzy rules and membership functions. If the resulting weight of a rule is close to zero, the rule can be safely removed from the rule base, since it is insignificant compared to others. Also, the shape and position of the membership functions in the Fuzzification and Defuzzification Layers can be fine tuned by adjusting the parameters of the neurons in these layers, during the training process.

## Simulation results

To show the contribution of the control by ANFIS and its improvements compared to the Neural Network method, simulation was approved on an unmanned vehicle.

**Figure 6:**
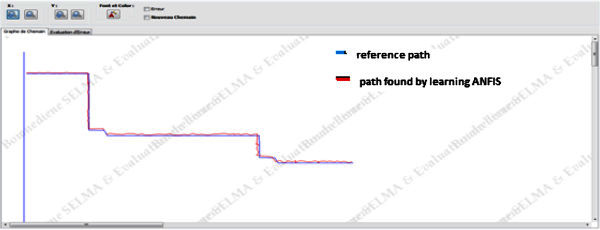
**The path found by ANFIS learning.**

Figure 6 shows the path followed by the UV controlled by the proposed ANFIS including obstacles described in the above sections.

**Figure 7:**
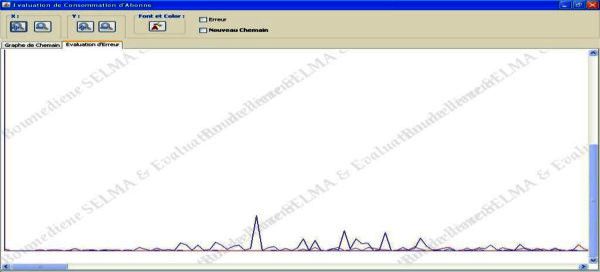
**The error between the reference path and the path calculated.**

As we can see from Figure 7 the path obtained from simulation setup is more close to the reference path which validates the proposed method.

**Figure 8:**
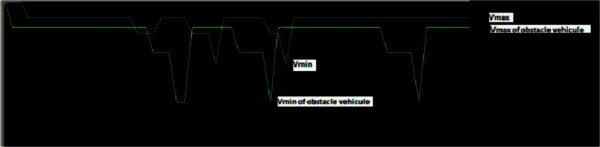
**UV speed variations at obstacles.**

The function of the Speed Controller subsystem is to achieve the desired speed that is to say the increase and decrease in speed on the road and especially in front of obstacles, so the accelerator control. Figure 8 shows the variation of the UV speed in function of obstacles come across the road.

From the above figure, it can be seen that the UV can indeed avoid obstacles and reach the targets. To verify the feasibility of proposed method Table 2 shows a comparison between neural network and ANFIS controller.

**Table 2:** **ANFIS-ANN results**

Method	Error	Success
ANFIS	0.79%	99,21%
ANN	1.95%	98.05%

### Comparison of the anfis results with other techniques

To show the performance of the results obtained by our approach, an artificial neural networks ANN have been selected for comparison because of its high capacity of prediction and control in non linear dynamical systems.

**Figure 9:**
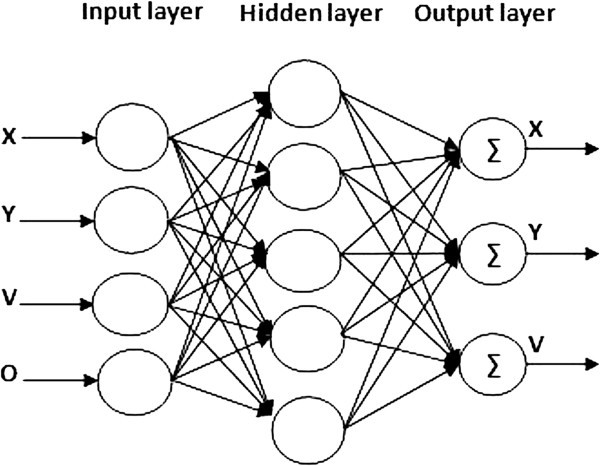
**ANN Architecture.**

Figure 9 shows the ANN used to control the UV,

From Table 2 it is seen the precision of the results of both methods are more or less the same. Therefore, it can be concluded that the ANFIS controller have a good potential to effect fast response to obstacles and reduce errors.

## Conclusion

ANFIS architecture has demonstrated a good performance in modeling the trajectory of an Unmanned Vehicle. The best feature of ANFIS is that it pre-processes all the data into several membership functions before mapping the data into an adaptive neuro structure. This pre processing feature allows ANFIS to converge faster and better. In order to be able to have a basis for comparison, an ANN type controller is also designed.

Through experimental tests and comparisons with existing algorithms, it is found that the proposed system is a powerful tool for controlling non linear dynamical systems.
